# Effects of fermented rice bran extract with multi-microbial species on intestinal health and growth of nursery pigs

**DOI:** 10.5713/ab.25.0402

**Published:** 2025-08-25

**Authors:** Lan Zheng, Jeonghyeon Son, Sung Woo Kim

**Affiliations:** 1 Department of Animal Science, North Carolina State University, Raleigh, NC, USA

**Keywords:** Fermented Rice Bran Extract, Growth Performance, Intestinal Health, Nursery Pig, Prebiotics, Probiotics

## Abstract

**Objective:**

The objective of this study was to investigate the effects of fermented rice bran extract (FRB) with multi-microbial species on intestinal health and growth performance of nursery pigs.

**Methods:**

Thirty weaned pigs (initial body weight = 6.8±0.8 kg) were allocated to 3 dietary treatments in a randomized complete block design (n = 10 per treatment) and fed for 25 d in 2 phases (7 and 18 d, respectively). Pigs were housed individually in pens equipped with a feeder and a nipple drinker. Pigs were fed a basal diet supplemented with 0%, 0.5%, or 1.0% FRB. The main feedstuffs of the basal diet were corn, soybean meal, whey permeate, and blood plasma. The FRB was prepared by fermenting rice bran with *Lactobacillus plantarum*, *Bacillus subtilis*, and *Saccharomyces cerevisiae*, and contained at least 1×10^7^ CFU/g. On d 25, pigs were euthanized to collect intestinal tissues and mucosa for intestinal health.

**Results:**

The supplementation of FRB decreased (p<0.05) the abundance of *Desulfovibrio piger* in the jejunal mucosa. Malondialdehyde and protein carbonyl in the duodenum linearly decreased (p<0.05) and the villus height to crypt depth ratio in the jejunum linearly increased (p<0.05) with increasing FRB supplementation. The apparent ileal digestibility of gross energy and crude protein tended to linearly increase (p = 0.084 and p = 0.098, respectively). Body weight on d 25 and overall average daily gain tended to show quadratic responses (p = 0.084 and p = 0.095, respectively) with increasing FRB supplementation. The gain to feed ratio (G:F) was maximized when the FRB intake was 2.7 g/d (0.48%) during d 7 to 25.

**Conclusion:**

Dietary supplementation with FRB with multi-microbial species improved intestinal health based on immune response, oxidative stress, and morphology. The growth performance of nursery pigs showed quadratic responses with increasing FRB supplementation. Specifically, the G:F was maximized with supplementation of FRB at 0.48%.

## INTRODUCTION

Nursery pigs are exposed to multiple stressors during weaning, including the transition from milk to solid diets, separation from the sow, mixing with unfamiliar pigs, and exposure to a new environment, making them more vulnerable to pathogens [1,2]. Additionally, the intestinal tract of nursery pigs is not fully developed during this period [2]. As a result, the intestinal health of nursery pigs may deteriorate, which can lead to diarrhea and growth retardation [1,3]. To mitigate these negative impacts, dietary interventions have been proposed to improve intestinal health and growth performance in nursery pigs [4–7]. Among the potential interventions, prebiotics and probiotics have been shown to improve the intestinal health of nursery pigs. Prebiotics, such as xylo-oligosaccharides and manno-oligosaccharides, are fermentable substrates that selectively stimulate proliferation of beneficial microorganisms [8,9]. On the other hand, probiotics are live microorganisms that positively modulate intestinal microbiome [10,11]. The microbiome, when beneficially modulated by prebiotics and probiotics, can reduce oxidative stress and unnecessary immune stimulation, improve intestinal morphology and nutrient digestibility, and ultimately growth performance of pigs [11–13].

Fermented rice bran extract (FRB) may act as a synbiotic, providing both prebiotic and probiotic effects that could improve the intestinal health and growth performance of nursery pigs. Fermentation of rice bran with multi-microbial species including *Lactobacillus plantarum*, *Bacillus subtilis*, and *Saccharomyces cerevisiae* could reduce the molecular size of non-starch polysaccharides (NSP) in the rice bran, providing the prebiotic effects [14–21]. During the fermentation, *Lactobacillus plantarum* can secrete β-glucosidase, hydrolyzing β-1,4 glycosidic bonds and leading to the production of oligosaccharides [17]. *Bacillus subtilis* can also hydrolyze polysaccharides into oligosaccharides by secreting NSP-degrading enzymes, such as cellulase, xylanase, and β-glucanase [18]. *Saccharomyces cerevisiae* does not hydrolyze polysaccharides due to the absence of NSP-degrading enzymes. However, co-fermentation by *Saccharomyces cerevisiae* and *Lactobacillus plantarum* can enhance the fiber-degrading capacity of *Lactobacillus plantarum* [22]. This effect may be attributed to a favorable environment created by *Saccharomyces cerevisiae*, such as a decrease in pH caused by the production of gas during fermentation, which supports the growth and enzymatic activity of *Lactobacillus plantarum* [4,23]. Based on this reason, the fermentation of multi-microbial species could enhance prebiotic effects by increasing oligosaccharides for lactic acid bacteria [24–27].

In addition to prebiotic effects, fermenting rice bran with *Lactobacillus plantarum*, *Bacillus subtilis*, and *Saccharomyces cerevisiae* could provide probiotic effects to nursery pigs because microorganisms used for fermentation remain viable in the product. Supplementation with *Lactobacillus plantarum* in nursery pig diets improved growth performance by positively modulating the intestinal microbiota and improving the intestinal morphology [28,29]. Similarly, supplementation with *Bacillus subtilis* and *Saccharomyces cerevisiae* has also been reported to enhance the growth performance of nursery pigs by positively modulating intestinal microbiota and improving intestinal morphology [4,30,31].

Based on these previously reported positive effects, it is hypothesized that supplementation with FRB in diets would positively modulate the mucosa-associated microbiota and reduce excessive immune response and oxidative stress in the jejunum supporting villus structure, improving nutrient digestibility, and finally enhancing growth of nursery pigs. To test these hypotheses, the objective of this study was to investigate the effects of FRB on intestinal health and growth performance of nursery pigs.

## MATERIALS AND METHODS

### Fermented rice bran extract

The FRB was a commercially available material that was obtained from Maxcell Global. Briefly, rice bran (*Oryza sativa*) medium was inoculated with a mixture of multi-microbial species (*Lactobacillus plantarum*, *Bacillus subtilis*, and *Saccharomyces cerevisiae*) in the presence of sucrose. The liquid suspension was centrifuged, and the supernatant was dried and ground into powder. The chemical composition of FRB was analyzed by North Carolina Department of Agriculture and Consumer Services Food and Drug Protection Division Laboratory (Table 1). Glycosyl composition and degree of polymerization of FRB were analyzed at Complex Carbohydrate Research Center of University of Georgia. The FRB contained at least 1×10^7^ CFU/g of *Lactobacillus plantarum*, *Bacillus subtilis*, and *Saccharomyces cerevisiae*, respectively.

### Animals, experimental design, and experimental diets

Thirty newly weaned pigs (15 barrows and 15 gilts) with an initial body weight of 6.8±0.8 kg (21-day-old) were randomly allocated to 3 dietary treatments in a randomized complete block design, using initial body weight and sex as blocks. Each treatment had 10 replicates (5 pens with barrows and 5 pens with gilts). Pigs were housed individually in pens (1.50 m× 0.74 m) equipped with a feeder and a nipple drinker, allowing them free access to both feed and water. The basal diets were formulated for phases 1 (d 0 to 7) and 2 (d 7 to 25) using corn, soybean meal, whey permeate, poultry meal, and blood plasma (Table 2). Zinc oxide was supplemented at 0.25% in the experimental diets. The basal diet was supplemented with FRB at 0.5% and 1.0% at the expense of corn and poultry fat. Each diet was formulated to meet or exceed the nutrient requirement estimated by NRC [32]. During the last 5 d of the experiment, titanium dioxide (0.5%) was added to experimental diets as an indigestible marker for determination of apparent ileal digestibility (AID) based on marker contents.

### Sample and data collection

The body weight of pigs and feed disappearance of each pen were measured on d 0, 7, and 25 to determine average daily gain (ADG), average daily feed intake (ADFI), and gain to feed ratio (G:F). On d 25, blood samples were collected from the jugular vein of pigs using BD Vacutainers. The fecal score of each pen was recorded every day throughout the experimental period, using a 1–3 scale: (1) very hard stool, (1.5) firm stool, (2) normal stool, (2.5) loose stool, and (3) watery stool with no shape. The collected samples were then centrifuged at 3,000×*g* for 15 min at 4°C to obtain serum. On d 25, pigs were euthanized using a captive bolt followed by exsanguination. Subsequently, tissues from the duodenum (5 cm after the pyloric-duodenal junction) and mid-jejunum (5 m after the pyloric-duodenal junction) were collected and flushed with a 0.9% sterile saline solution. Ileal digesta was collected from 30 cm anterior to the ileocecal junction in a 100-mL container and put on the ice, then stored at −20°C for measurement of AID of gross energy (GE) and nutrients following Deng et al [14] and Chen et al [33]. The mucosa were scraped from the first 15 cm of the collected duodenum and jejunum, then immediately frozen in liquid nitrogen and stored at −80°C. The remaining parts of the duodenum and jejunum were fixed in a 10% buffered formalin solution. Ileal digesta were stored in a sterile container and kept frozen at −20°C.

### Differential abundance of the mucosa-associated microbiota in the jejunum

The QIAamp DNA Stool Mini Kit (#51504; Qiagen) was used to extract DNA from the collected jejunal mucosa. The extracted DNA samples were sent to MAKO Medical Laboratories for qPCR analysis of 16S rDNA sequences. The Ion Chef instrument and Ion S5 system were used to prepare samples for template sequencing, respectively. The Ion 16S Metagenomics Kit (A26216; Thermo Fisher Scientific) was utilized to amplify the variable regions (V2, V3, V4, V6, V7, V8, and V9) of the 16S rRNA gene. The Ion Xpress Plus Fragment Library Kit (Cat. 4471269; Thermo Fisher Scientific) was employed to create libraries from the amplified regions, along with the Ion Code Barcode Adapters 1–384 Kit (A29751; Thermo Fisher Scientific). The Ion Universal Library Quantitation Kit (A26217; Thermo Fisher Scientific) was utilized to quantify the libraries. The Torrent Suite Software (ver. 5.2.2) was employed to process the sequences, generating unaligned bam files for subsequent analysis. The Ion Reporter Software Suite (ver. 5.2), a set of bioinformatics analysis tools, was utilized for sequence data analysis and alignment with the GreenGenes and MicroSeq databases, as well as for generating alpha and beta diversity plots and OTU tables. All samples exhibited sequencing coverage depth exceeding 1,000×.

### Immune responses and oxidative stresses status

One gram of the collected mucosa samples was homogenized using a homogenizer (Tissuemiser; Thermo Fisher Scientific) to analyze levels of immunoglobulin A (IgA), immunoglobulin G (IgG), tumor necrosis factor-α (TNF-α), malondialdehyde (MDA), and protein carbonyl (PC). The supernatant was collected after centrifuging the homogenized mucosa at 14,000 g for 30 min at 4°C. The total protein content of the supernatant was measured using the pierce BCA Protein Assay kit (23225#; Thermo Fisher Scientific) following the procedure described by Deng et al [14]. The measured total protein concentration was used to standardize the concentration of the immune response criteria. The duodenal and jejunal mucosa were analyzed using ELISA kits for pig IgA (E101–102) and pig IgG (E101–104) from Bethyl Laboratories, respectively. The contents of TNF-α in mucosa were determined using ELISA kits (R&D Systems) following the method described by Jang and Kim [34]. The mucosal MDA and PC contents were measured using commercial assay kits (Cell Biolabs) following the method described by Choi et al [35]. The serum concentrations of IgG, TNF-α, MDA, and PC were also analyzed using the same methods used for the mucosa.

### Intestinal morphology

The collected duodenal and jejunal tissues were used to evaluate intestinal morphology. After 48 h of fixation in 10% buffered formaldehyde, two sections of the tissues (approximately 2 mm each) were cut, placed in a cassette, and transferred to a 70% ethanol solution. The processed samples were then sent to the North Carolina State University Histology Laboratory (College of Veterinary Medicine) for dehydration, embedding, and staining. Hematoxylin and eosin staining was used to evaluate jejunal morphology, including villus height (VH) and crypt depth (CD). Ki-67^+^ proliferative cells were stained using 3,3’-diaminobenzidine.

The microscope Olympus CX31 (Lumenera Corporation) was used to measure VH and CD. The pictures were taken at magnification of 40× using a digital camera (Infinity 2-2 digital CCD; Lumenera Corporation) and analyzed using a software (Infinity Analyze microscopy software; Lumenera). Ten intact villi and associated crypts in each slide were measured as described by Deng et al [14]. The VH was measured from the top of the villi to the junction of villi and crypt, whereas CD was measured from the bottom of the crypt to the junction of villi and crypt. The ratio of VH to CD (VH:CD) was calculated by dividing VH by CD.

Pictures of the jejunal crypts were taken at 100× magnification to calculate the proportion of Ki-67^+^ proliferative cells in the crypt. The percentage of Ki-67^+^ proliferative cells was determined by dividing the number of Ki-67^+^ proliferative cells by the total number of cells in the crypts [36]. Cell counting was performed using ImageJS software. All morphological analyses were conducted by the same person, and the average of 15 measurements per slide was calculated and reported as one observation.

### Chemical analyses

Frozen ileal digesta were freeze-dried in a freeze drier. Experimental diets and dried ileal digesta were finely ground and analyzed for dry matter (DM; method 930.15) as described in AOAC International [37]. Nitrogen contents in the diets and ileal digesta were measured using a TrueSpec N Nitrogen Determinator (LECO) to calculate crude protein (CP; 6.25×nitrogen). Experimental diets and ileal digesta were analyzed for GE using a bomb calorimetry (Parr 1261; Parr Instrument). Amylase and protease activities in FRB were determined using the methods described by Bernfeld [38] and Anson [39], respectively. One unit of amylase activity was defined as the amount of enzyme that releases 1 μmol of glucose equivalent from a water-insoluble, cross-linked starch polymer substrate per 1 mit at pH 6.5 and 37°C [38]. One unit of protease activity was defined as the amount of enzyme that liberates 1μg of tyrosine per 1 min at pH 3.0 and 40°C [39]. The titanium dioxide concentrations of the diets and ileal digesta were determined [40]. Briefly, the samples were digested in Kjeldahl digestion tubes with a catalyst and 13 mL of concentrated sulfuric acid at 420°C for 2 h. After cooling for 30 min, 10 mL of 30% hydrogen peroxide was added each tube, and total liquid volume was adjusted to 100 mL with distilled water. The liquid was transferred to a microplate to determine TiO_2_ at 410 nm using spectrophotometry.

### Apparent ileal digestibility

The AID of nutrients was calculated according to the following equation [41]:


(1) $$\text{AID of Nutr }(\%)=(1-[{\text{Ti}}_{\text{diet}}÷{\text{Ti}}_{\text{digesta}}]\times [{\text{Nutr}}_{\text{digesta}}÷{\text{Nutr}}_{\text{diet}}])\times 100$$

Where Ti_diet_ and Ti_digesta_ are the TiO_2_ contents in the diet and ileal digesta, respectively (%; DM basis); and Nutr_digesta_ and Nutr_diet_ are the nutrient concentration in the ileal digesta and diet, respectively (%; DM basis). The AID of GE was also calculated using the same equation. The GE concentrations in the diet and ileal digesta were expressed as kcal/kg DM.

### Statistical analyses

Data were analyzed using the MIXED procedure of SAS 9.4 (SAS Institute). Diet was treated as a fixed effect, whereas body weight and sex were included as random effects. Least squares mean for each treatment was calculated. The effects of increasing levels of FRB in the diets of nursery pigs were determined using the polynomial contrasts (linear and quadratic effects) with coefficients generated by the IML procedure of SAS. The analysis of similarities was performed to evaluate the beta diversity of mucosa-associated microbiota using the ANOSIM procedure of SAS. The data were visualized using principal coordinates analysis based on Bray-Curtis distance. The differential abundance of each microbiota at the family, genus, and species levels among experimental diets was analyzed using linear discriminant analysis (LDA) Effect Size in R (ver. 4.4.1) following Cheng et al [42] and Gormley et al [43]. The analysis was performed using microbiomeMarker package (ver. 1.12.2). The group variable was diet, and taxa with an LDA score greater than 2.0 and a p-value less than 0.05 were considered significantly different.

To reflect variation in FRB intake (g/d) within the same supplementation level, dose-response modeling was conducted based on the FRB intake. Quadratic and one-slope broken-line models were developed using the NLIN procedure of SAS to determine the key level of FRB intake affecting growth performance and oxidative stress. The FRB intake was calculated by multiplying ADFI (g/d) by dietary supplementation level of FRB (%). The FRB intake was used as the independent variable, whereas growth performance and oxidative stress were used as dependent variables. Due to the low feed intake and body weight gain mainly caused by weaning stress during phase 1 (d 0 to 7), the models in response to FRB intake were developed using only phase 2 (d 7 to 25) data. The estimated optimal levels or break points of FRB intake from developed models were then converted into FRB level (%) based on the ADFI (568 g/d) measured during phase 2. Only models showing statistical significance or a tendency were presented. The experimental unit was a pig. Significance and tendency were declared at p<0.05 and 0.05≤p<0.10, respectively.

## RESULTS

**Figure 1:**
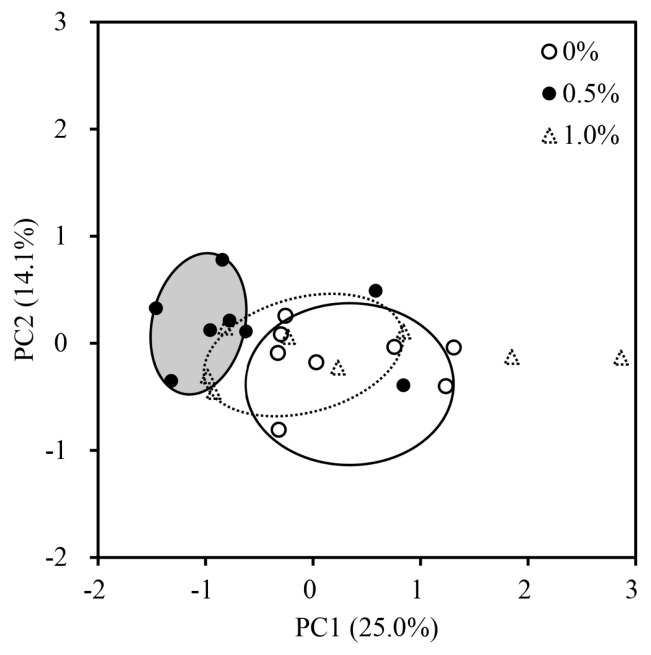
Principal coordinates analysis of the jejunal mucosa-associated microbiota at genus level of nursery pigs fed diets with 0%, 0.5% or 1.0% fermented rice bran extract (FRB). The X-axis and Y-axis represent the principal component axes, with the percentages indicating the proportion of variation explained by each component. Each symbol represents a pig: open circles indicate 0% FRB group, filled circles indicate 0.5% FRB group, and open triangles with dotted lines indicate 1.0% FRB group. Ellipses denote 95% confidence intervals for each group: open circle with solid line for 0%, gray-filled circle with solid line for 0.5%, and open circle with dotted line for 1.0%. The beta diversity value of nursery pigs fed 0.5% FRB diet was different (p<0.05) from that of nursery pigs fed 0% or 1.0% FRB diet. The number of observations was 30.

**Figure 2:**
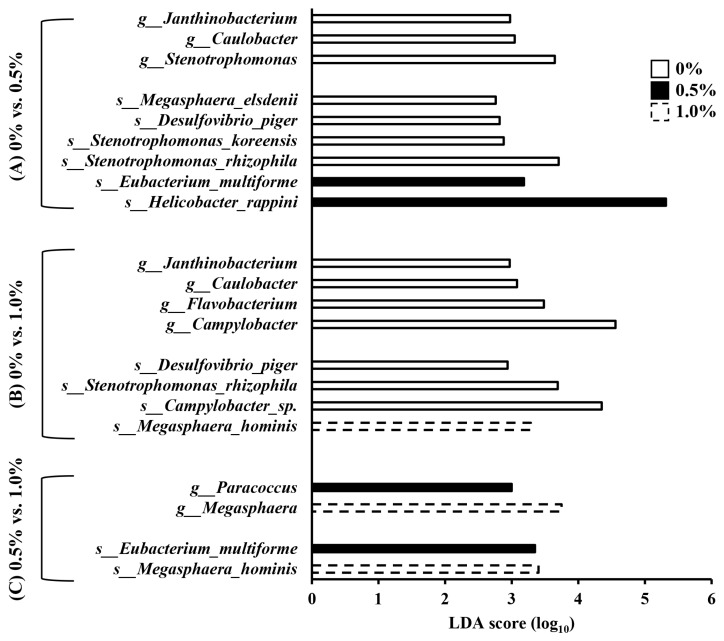
Linear discriminant analysis (LDA) effect size of jejunal mucosa-associated microbiota at genus and species levels of nursery pigs fed diets with 0%, 0.5%, or 1.0% fermented rice bran extract (FRB). Three panels represent the pairwise comparisons between dietary treatments: (A) 0% vs. 0.5%; (B) 0% vs. 1.0%; (C) 0.5% vs. 1.0%. The X-axis and Y-axis represent the LDA score and bacterial taxa (genus and species), respectively. The number of observations for each group was 10. The grouping variable was diet, and taxa with an LDA score greater than 2.0 and a p-value less than 0.05 were considered significantly different. No significant different taxa were identified at the family level.

**Figure 3:**
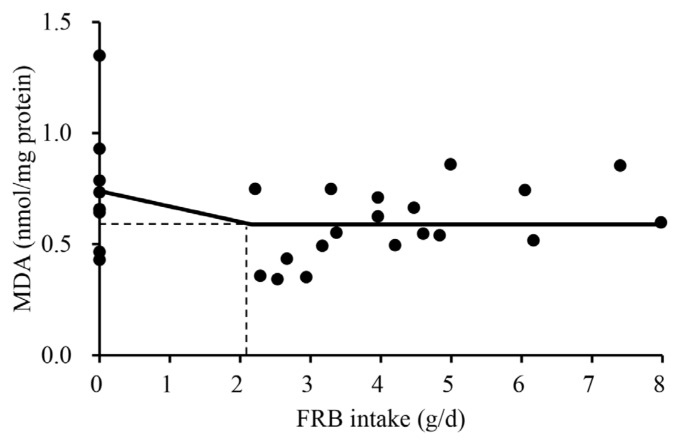
A one-slope broken line model of the malondialdehyde (MDA) level in the duodenal mucosa of nursery pigs with increasing daily intake (g/d) of fermented rice bran extract (FRB). A one-slope broken line model of the MDA indicated that the daily intake of FRB required for reaching plateau of MDA levels (0.59 nmol/mg protein) was 2.2 g/d. Break point was estimated based on the following equation: Y = 0.59+0.070×(2.2–X) where X is less than 2.2 (p<0.10). The FRB level required to reach the plateau of MDA reduction in nursery pigs was 0.39%, based on the average daily feed intake (568 g/d) during phase 2 (d 7 to 25). The experimental unit was a pig, and the number of observations was 30.

**Figure 4:**
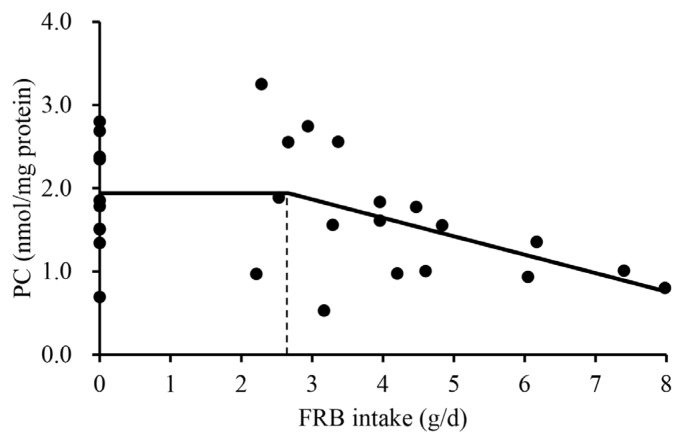
A one-slope broken line model of the protein carbonyl (PC) level in the duodenal mucosa of nursery pigs with increasing daily intake (g/d) of fermented rice bran extract (FRB). A one-slope broken line model of the PC indicated that the daily intake of FRB required to initiate a decrease in PC levels (1.9 nmol/mg protein) was 2.7 g/d. Break point was estimated based on the following equation: Y = 1.9+0.022×(2.7–X) where X is greater than 2.7 (p<0.05). The minimum effective level of FRB required to reduce PC was 0.48%, based on the average daily feed intake (568 g/d) during phase 2 (d 7 to 25). The experimental unit was a pig, and the number of observations was 30.

**Figure 5:**
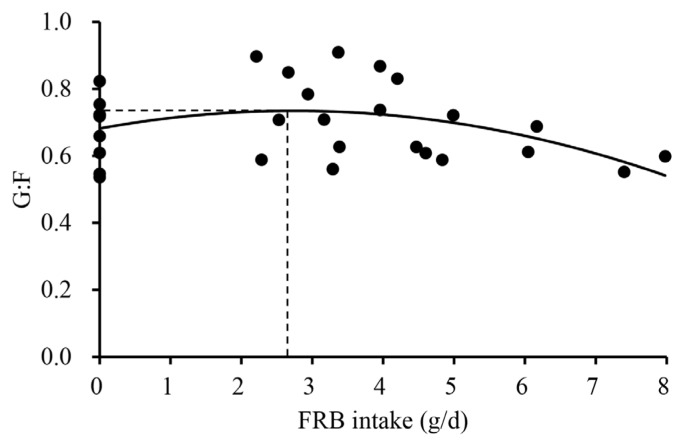
A quadratic model of the gain to feed ratio (G:F) of nursery pigs with increasing daily intake (g/d) of fermented rice bran extract (FRB). A quadratic model of G:F indicated that the daily intake of FRB required for maximizing G:F (0.73) was 2.7 g/d. Maximum point was estimated based on the following equation: Y = 0.73–0.0070×(2.7–X)^2^ where X is 2.7 (p<0.10). The optimal FRB level required for maximizing G:F was 0.48%, based on the average daily feed intake (568 g/d) during phase 2 (d 7 to 25). The experimental unit was a pig, and the number of observations was 30.

Effects of increasing FRB supplementation on alpha diversity were not observed (Table 3). The overall jejunal mucosa-associated microbiota community of nursery pigs fed the 0.5% FRB diet was different (p<0.05) from those of pigs fed either 0% or 1.0% FRB diets (Figure 1). In the comparison between 0% and 0.5% FRB diets, *Janthinobacterium*, *Caulobacter*, and *Stenotrophomonas* were enriched (p<0.05) in the jejunal mucosa of pigs fed the 0% FRB diet at the genus level (Figure 2). At the species level, *Megasphaera elsdenii*, *Desulfovibrio piger*, *Stenotrophomonas koreensis*, and *Stenotrophomonas rhizophila* were enriched (p<0.05) in the jejunal mucosa of pigs fed the 0% FRB diet, whereas *Eubacterium multiforme* and *Helicobacter rappini* were enriched (p<0.05) in the jejunal mucosa of pigs fed the 0.5% FRB diet. In the comparison between 0% and 1.0% FRB diets, *Janthinobacterium*, *Caulobacter*, *Flavobacterium*, and *Campylobacter* were enriched (p<0.05) in the jejunal mucosa of pigs fed the 0% FRB diet at the genus level. At the species level, *Desulfovibrio piger*, *Stenotrophomonas rhizophila*, and *Campylobacter* sp. were enriched (p<0.05) in the jejunal mucosa of pigs fed the 0% FRB diet, whereas *Megasphaera hominis* was enriched (p<0.05) in the pigs fed the 1.0% FRB diet. In the comparison between 0.5% and 1.0% FRB diets, *Paracoccus* was enriched (p<0.05) in the jejunal mucosa of pigs fed the 0.5% FRB diet, whereas *Megasphaera* was enriched (p<0.05) in the pigs fed the 1.0% FRB diet at the genus level. At the species level, *Eubacterium multiforme* was enriched (p<0.05) in the jejunal mucosa of pigs fed the 0.5% FRB diet, whereas *Megasphaera hominis* was enriched (p< 0.05) in the pigs fed the 1.0% FRB diet. Serum IgG quadratically increased (p<0.05) and then decreased with increasing FRB supplementation (Table 4). Duodenal mucosa IgG tended to linearly increase (p = 0.058) with increasing FRB supplementation. Duodenal mucosa MDA and PC linearly decreased (p<0.05) with increasing FRB supplementation. The PC in jejunal mucosa linearly decreased (p = 0.094) with increasing FRB supplementation. Based on the broken-line model, MDA level in the duodenal mucosa decreased (0.74 to 0.59) until FRB intake increased from 0 to 2.2 g/d during phase 2 (Figure 3). Based on the ADFI (568 g/d) during phase 2, the FRB level required to reach the plateau of MDA reduction in nursery pigs was 0.39%. Based on the broken-line model, PC level in the duodenal mucosa began to decrease after FRB intake reached 2.7 g/d during phase 2 (Figure 4). Similarly, the minimum level of FRB required to initiate the reduction of PC is 0.48% during phase 2. The jejunal VH:CD linearly increased (p<0.05) with increasing FRB supplementation (Table 5). The Ki-67^+^ proliferative cell quadratically increased and then decreased with increasing FRB supplementation. The AID of DM (p = 0.099), GE (p = 0.084), and CP (p = 0.098) tended to linearly increase with increasing FRB supplementation (Table 6). The fecal score linearly decreased (p<0.05) from d 0 to d 7 and over the entire experimental period with increasing FRB supplementation (Table 7). The ADFI quadratically increased (p<0.05) from d 0 to d 7 with increasing FRB supplementation (Table 8). Based on the quadratic model, the G:F of nursery pigs increased (from 0.68 to 0.73) until the FRB intake reached 2.7 g/d, after, and then decreased (Figure 5). Based on the ADFI (568 g/d) during phase 2, the FRB level required to optimize the G:F of nursery pigs was 0.48%.

## DISCUSSION

In this study, supplementation with FRB did not affect the alpha diversity of the jejunal mucosa-associated microbiota, whereas 0.5% of FRB supplementation had a different beta diversity value compared with 0% and 1.0% of FRB supplementation. These results indicate that the composition of microbiota differs between diets without the change of richness and evenness in microbiome communities. These changes may be attributed to shifts in the abundance of each microbial taxa, rather than to the presence or absence of specific species. Furthermore, the confidence ellipse for pigs fed the 0.5% FRB diet appeared smaller than that for pigs fed the 0% or 1.0% FRB diet, suggesting that 0.5% FRB induced more consistent and less variable changes in the jejunal mucosa-associated microbiota composition.

The inclusion of FRB reduced the differential abundance of *Desulfovibrio piger* regardless of the FRB level, whereas *Eubacterium multiforme* increased at 0.5% and *Megasphaera hominis* increased at 1.0% FRB. The bacteria of *Desulfovibrio* genus are sulfate-reducing bacteria that produce hydrogen sulfide [44], whereas *Eubacterium* and *Megasphaera* genus are known to produce organic acids, including short-chain fatty acids (SCFA) and valeric acid [45,46]. These changes in species may have partially contributed to the intestinal health of pigs via microbial metabolites. However, in this study, the relative abundance of taxa affected by FRB supplementation was low (<2.0%) and functional information of these species was not available. For these reasons, the potential impacts of FRB on jejunal mucosa-associated microbiota of pigs should be interpreted with caution.

Increased IgG level in the duodenal mucosa may be associated with enhanced production of SCFA, such as acetate and butyrate, in response to FRB supplementation. The oligosaccharides present in FRB could serve as substrates for jejunal mucosa-associated microbiota, potentially increasing SCFA production by facilitating their metabolic activity without altering microbial composition. IgG is produced by plasma cells differentiated from activated B cells, and both antigen stimulation and SCFA produced by fiber-degrading bacteria can support this activation [47–49]. SCFA can serve as energy sources for B cells, indirectly supporting their activation and differentiation, or can bind to B cell receptors to directly promote differentiation [49–51].

MDA, a product of lipid peroxidation, is a well-known indicator of oxidative damage [52]. The reduced MDA in the duodenum and the jejunum was observed in this study and may be attributed to probiotics introduced via FRB. A recent study [53] reported that multi-strain probiotics increased total antioxidant capacity and superoxide dismutase activity because probiotics can produce antioxidant enzymes. A similar observation was reported by a previous study [54], in which supplementation of lactic acid bacteria neutralized free radicals by producing both enzymatic and non-enzymatic antioxidants. However, no antioxidant effects of FRB were observed in the serum. The reason for this discrepancy may be due to the fact that the reduction in oxidative stress induced by FRB is primarily originated from microbial antioxidants, and therefore, the effect is likely to be localized within the intestinal environment. In a similar manner, a reduction in mucosal PC, a kind of protein oxidation product, in the duodenum aligns with previous finding [10], which can also be explained by antioxidant effects of FRB. To investigate the changes in oxidative stress in response to FRB intake, one-slope broken line analyses were conducted. Although both MDA and PC are oxidative stress markers, the reduction of MDA occurred at a lower level of FRB intake than that in PC. Lipid peroxidation responds to oxidative stress readily further producing reactive oxygen species resulting in oxidative damages to proteins and DNA [55,56].

In this study, dietary supplementation with FRB failed to induce an improvement in VH or CD individually, likely due to the pharmacological inclusion of zinc oxide in the diets. Zinc oxide may have inhibited the proliferation of opportunistic pathogenic bacteria through the antimicrobial effects [5]. This inhibition may have minimized villus atrophy induced by dysbiosis, thereby limiting the potential for further modulation by dietary FRB. However, despite the lack of individual improvements, the VH:CD was improved with FRB supplementation. Interestingly, this finding is not uncommon and has also been reported in a previous study [35]. This finding indicates that combined minor changes in VH and CD can cumulatively lead to improvements in the VH:CD. Additionally, the reduction in oxidative damage, resulting from probiotics introduced via FRB, may have partially contributed to the improvement in the VH:CD. The quadratic response was observed in the proportion of Ki-67^+^ proliferative cells in the crypts as the level of FRB supplementation increased. As an indicator of cell proliferation, the increased proportion of Ki-67^+^ proliferative cells suggests FRB supplementation enhanced regeneration of villus epithelial cells [36,43]. Despite the absence of infections and external challenges, intestinal epithelial cells of pigs are continuously subjected to damage [57,58]. The FRB supplementation may help maintain epithelial resilience in the face of such ongoing damages.

The improvement in AID of GE and nutrients by dietary supplementation with prebiotics and probiotics has intensively been reported [10,13,59–62]. Several mechanisms may explain this improvement. First, modulation of the intestinal microbiota may have contributed to nutrient degradation, as specific bacteria can secrete microbial digestive enzymes that hydrolyze nutrients in digesta [62]. Second, an improved intestinal morphology, particularly an increased VH:CD, may have increased the absorptive surface area, thereby improving nutrient absorption. Third, microbial enzymes, such as protease and α-amylase, produced during the fermentation process of FRB may support the digestion of feed and feedstuffs.

A linear decrease in fecal score was observed during phase 1 and the overall period as the FRB supplementation increased. This observation aligns with previous studies reporting that dietary supplementation with probiotics and prebiotics decreased fecal score and incidence of diarrhea in nursery pigs [63,64]. Additionally, the reduction in sulfate-reducing bacterium and the increase in SCFA-producing bacteria with FRB supplementation may be associated with the linear decrease in fecal score. These microbial changes may have alleviated mucosal inflammation and supported the regulation of electrolyte balance in the jejunum, thereby contributing to the reduction in fecal scores [65].

In this study, a quadratic response in ADG and ADFI was observed as the supplemental levels of FRB increased. The initial improvement in growth performance, at 0.5% of FRB, can be explained by positive changes of the mucosa-associated microbiota in the jejunum, enhanced immune function, and increased digestibility, as discussed previously. This result can be supported by the estimated optimal level of FRB for G:F was 0.48% based on the developed model. However, FRB supplementation above 0.48% resulted in decreased growth performance. Interestingly, similar quadratic responses in growth performance have also been documented in previous studies using prebiotics [66] and probiotics [67,68], where excessive supplementation led to decreased growth performance of pigs. For example, Xing et al [66] reported that gradually increasing galacto-oligosaccharide from 0% to 0.2% in nursery pig diets resulted in a quadratic response in ADG and ADFI. Likewise, Lee et al [68] observed a quadratic response in ADFI in growing-finishing pigs supplemented with microbial additives consisting of *Lactobacillus plantarum*, *Bacillus subtilis*, *Bacillus amyloliquefaciens*, and *Saccharomyces cerevisiae*. In this study, the quadratic pattern observed in growth performance may be associated with microbial shifts because a distinct separation in the beta diversity of the 0.5% FRB group was observed compared to the 0% and 1.0% groups. *Eubacterium multiforme* was more abundant in pigs fed the 0.5% FRB diet compared to those fed the 0% or 1.0% FRB diets, and may be one of the taxa contributing to the distinct separation of beta diversity. Although its specific role in pigs has not been well reported, the members of the genus *Eubacterium* are known to produce various organic acids [45]. These results suggest this species may be associated with growth performance of pigs.

In this study, although the intestinal health criteria of nursery pigs were linearly improved with increasing FRB, growth performance exhibited a quadratic pattern with improvement at 0.5% FRB and a decline at 1.0% FRB. These findings suggest that the effects of FRB vary depending on the response criteria, and that the inclusion level of FRB should be determined based on the specific objective of supplementation. This interpretation is supported by the fact that the optimal levels of FRB differed between MDA and G:F.

All experimental diets in this study were supplemented with zinc oxide at pharmacological levels because this supplementation is adopted in nursery pig diet in the United States to prevent post-weaning diarrhea through its antimicrobial effects [5,58]. Therefore, the inclusion of zinc oxide in this study reflects a practical nursery pig diet. Zinc oxide is known to produce reactive oxygen species which can induce oxidative stress in bacteria [5]. This mode of action contrasts with the primary mechanisms of FRB and may have masked the effects of FRB. Despite the presence of zinc oxide, FRB supplementation altered microbiota composition, reduced oxidative damage, and improved jejunal morphology. These results suggest that although zinc oxide effectively prevents post-weaning diarrhea through its antimicrobial actions, there remains room to enhance other response criteria related to intestinal health. Such improvements may be achieved through the use of synbiotic additives like FRB. Moreover, these beneficial effects may become more pronounced when the pharmacological use of zinc oxide is limited.

## CONCLUSION

In conclusion, dietary supplementation with FRB changed the jejunal mucosa-associated microbiota composition, improved immune response, relieved oxidative stress, increased GE and nutrient digestibility, and enhanced intestinal morphology of nursery pigs. Although these improvements contributed to enhancing the intestinal health, the growth performance of nursery pigs showed quadratic responses with increasing FRB supplementation. The G:F of nursery pigs can be maximized with supplementation of FRB at 0.48%.

## Figures and Tables

**Table 1:** Chemical and glycosyl composition, degree of polymerization, and enzyme activity of fermented rice bran extract (as-is basis)

Item	Fermented rice bran extract
Chemical composition (%)	
Dry matter	97.64
Total carbohydrate	43.00
Crude protein	12.20
Crude fat	16.20
Crude fiber	9.10
Ash	8.00
Glycosyl composition (%)	
Glucose	98.00
Xylose	0.95
Arabinose	0.80
Mannose	0.45
Degree of polymerization (% carbohydrate)	
2	19.3
3	48.6
4	27.6
5	3.5
6	0.9
Enzyme activity (unit/g)	
α-amylase^1)^	13.2
Protease^2)^	12.1

1) One unit of amylase activity was defined as the amount of enzyme that releases 1 μmol of glucose equivalent from a water-insoluble, cross-linked starch polymer substrate per 1 unit at pH 6.5 and 37°C [38].

2) One unit of protease activity was defined as the amount of enzyme that liberates 1 μg of tyrosine per 1 min at pH 3.0 and 40°C [39].

**Table 2:** Feedstuffs and chemical composition of experimental diets (as-fed basis)

Item	Phase 1 (d 0 to 7)			Phase 2 (d 7 to 25)		
	
	0% FRB	0.5% FRB	1.0% FRB	0% FRB	0.5% FRB	1.0% FRB
Feedstuff (%)						
Corn (yellow dent)	41.50	41.18	40.86	52.92	52.60	52.28
Soybean meal	20.00	20.00	20.00	24.50	24.50	24.50
Whey permeate	20.00	20.00	20.00	10.00	10.00	10.00
Poultry meal	6.00	6.00	6.00	3.00	3.00	3.00
Blood plasma	7.00	7.00	7.00	3.00	3.00	3.00
FRB	0.00	0.50	1.00	0.00	0.50	1.00
Poultry fat	2.50	2.36	2.23	3.30	3.16	3.03
L-Lys·HCl (78.8%)	0.45	0.45	0.45	0.46	0.46	0.46
DL-Met (99.0%)	0.22	0.22	0.22	0.18	0.18	0.18
L-Thr (98.0%)	0.15	0.15	0.15	0.13	0.13	0.13
Limestone	1.30	1.30	1.30	0.94	0.94	0.94
Dicalcium phosphate	0.20	0.20	0.20	0.92	0.92	0.92
Vitamin premix^1)^	0.03	0.03	0.03	0.03	0.03	0.03
Mineral premix^2)^	0.15	0.15	0.15	0.15	0.15	0.15
Sodium chloride	0.25	0.25	0.25	0.22	0.22	0.22
Zinc oxide	0.25	0.25	0.25	0.25	0.25	0.25
Calculated composition						
Dry matter (%)	91.5	91.1	90.7	90.2	89.8	89.4
Metabolizable energy (kcal/kg)	3,440	3,435	3,430	3,456	3,451	3,447
Crude protein (%)	23.7	23.7	23.6	21.2	21.2	21.2
SID Lys (%)	1.51	1.51	1.51	1.35	1.35	1.35
SID Met+Cys (%)	0.83	0.83	0.83	0.74	0.74	0.74
SID Trp (%)	0.26	0.26	0.26	0.22	0.22	0.22
SID Thr (%)	0.89	0.89	0.89	0.79	0.79	0.78
Calcium (%)	0.86	0.86	0.86	0.80	0.80	0.80
STTD phosphorus (%)	0.46	0.46	0.46	0.40	0.40	0.40
Analyzed composition						
Gross energy (kcal/kg)	-	-	-	3,973	4,015	4,004
Crude protein (%)	-	-	-	22.0	23.0	22.0

1) The vitamin premix provided the following per kilogram diet: 3,968 IU of vitamin A; 1,190 IU of vitamin D_3_; 20 IU of vitamin E; 0.012 mg of vitamin B12; 4.0 mg of riboflavin; 33 mg of niacin; 6.6 mg of d-pantothenic acid; 1.2 mg of menadione; 0.012 IU of biotin.

2) The mineral premix provided the following per kilogram diet: 17 mg of Cu; 0.297 mg of I; 110 mg of Fe; 33 mg of Mn; 0.297 mg of Se; 110 mg of Zn.

FRB, fermented rice bran extract; SID, standardized ileal digestible; STTD, standardized total tract digestible.

**Table 3:** Alpha diversity of jejunal mucosa-associated microbiota at the genus level in nursery pigs fed diets with increasing levels of fermented rice bran extract

Item	Fermented rice bran extract (%)			SEM	p-value	
	
	0	0.5	1.0		Linear	Quadratic
Chao1	67.4	69.3	70.9	10.0	0.366	0.823
Shannon	2.91	3.29	3.21	0.26	0.520	0.583
Simpson	0.74	0.81	0.80	0.03	0.400	0.524

Each least squares mean represents 10 observations.

SEM, standard error of the mean.

**Table 4:** Immune responses and oxidative damages in the serum and intestinal mucosa of nursery pigs fed diets with increasing levels of fermented rice bran extract

Item	Fermented rice bran extract (%)			SEM	p-value	
	
	0	0.5	1.0		Linear	Quadratic
Serum (unit/mL)						
Immunoglobulin G (μg)	1.04	1.42	1.12	0.14	0.669	0.036
Tumor necrosis factor-α (pg)	15.7	15.9	17.1	1.0	0.351	0.714
Malondialdehyde (nmol)	6.61	8.32	8.87	1.31	0.255	0.693
Protein carbonyl (nmol)	0.66	0.61	0.64	0.09	0.897	0.625
Duodenum (unit/mg protein)						
Immunoglobulin A (μg)	2.40	3.48	2.29	0.57	0.974	0.194
Immunoglobulin G (μg)	1.64	1.91	2.10	0.18	0.058	0.864
Tumor necrosis factor-α (μg)	1.19	0.80	0.95	0.17	0.258	0.185
Malondialdehyde (nmol)	0.75	0.58	0.61	0.06	0.049	0.068
Protein carbonyl (nmol)	1.89	1.90	1.26	0.21	0.035	0.214
Jejunum (unit/mg protein)						
Immunoglobulin A (μg)	1.13	1.30	1.35	0.11	0.208	0.689
Immunoglobulin G (μg)	1.71	1.68	1.69	0.18	0.964	0.955
Tumor necrosis factor-α (μg)	1.15	1.00	0.88	0.13	0.180	0.903
Malondialdehyde (nmol)	0.52	0.43	0.52	0.08	0.912	0.318
Protein carbonyl (nmol)	1.92	1.93	1.53	0.21	0.094	0.353

Each least squares mean represents 10 observations.

SEM, standard error of the mean.

**Table 5:** Intestinal morphology and crypt cell proliferation in the duodenum and jejunum of nursery pigs fed diets with increasing levels of fermented rice bran extract

Item	Fermented rice bran extract (%)			SEM	p-value	
	
	0	0.5	1.0		Linear	Quadratic
Duodenum						
Villus height (μm)	551	557	526	24	0.503	0.592
Villus width (μm)	94	95	101	5	0.341	0.402
Crypt depth (μm)	305	295	281	16	0.286	0.827
Villus height-to-crypt depth ratio	1.8	1.9	1.9	0.1	0.520	0.715
Jejunum						
Villus height (μm)	452	445	468	19	0.496	0.456
Villus width (μm)	74	73	72	2	0.659	0.954
Crypt depth (μm)	214	205	197	8	0.193	0.915
Villus height-to-crypt depth ratio	2.1	2.2	2.4	0.1	0.018	0.501
Ki-67^+^ proliferative cell (%)^1)^	15.4	18.4	15.8	1.2	0.775	0.043

Each least squares mean represents 10 observations.

1) Ratio of Ki-67+ proliferative cell to total cell in the crypt, which represents crypt cell proliferation.

SEM, standard error of the mean.

**Table 6:** Apparent ileal digestibility of nutrients and gross energy in nursery pigs fed diets with increasing levels of fermented rice bran extract

Item	Fermented rice bran extract (%)			SEM	p-value	
	
	0	0.5	1.0		Linear	Quadratic
Apparent ileal digestibility (%)						
Dry matter	68.0	69.5	71.1	1.3	0.099	0.957
Gross energy	68.4	70.0	71.6	1.4	0.084	0.920
Crude protein	76.9	78.7	80.3	1.8	0.098	0.921

Each least squares mean represents 10 observations.

SEM, standard error of the mean.

**Table 7:** Fecal score of nursery pigs fed diets with increasing levels of fermented rice bran extract

Item	Fermented rice bran extract (%)			SEM	p-value	
	
	0	0.5	1.0		Linear	Quadratic
Fecal score^1)^						
Phase 1 (d 0 to 7)	1.23	1.09	1.09	0.05	0.026	0.245
Phase 2 (d 7 to 25)	1.03	1.00	1.00	0.01	0.090	0.213
Overall (d 0 to 25)	1.09	1.03	1.03	0.02	0.013	0.125

Each least squares mean represents 10 observations.

1) 1, very hard and dry stool; 1.5, firm stool; 2, normal stool; 2.5, loose stool; 3, watery stool with no shape.

SEM, standard error of the mean.

**Table 8:** Growth performance of nursery pigs fed diets with increasing levels of fermented rice bran extract

Item	Fermented rice bran extract (%)			SEM	p-value	
	
	0	0.5	1.0		Linear	Quadratic
Body weight (kg)						
d 0	6.8	6.7	6.5	0.4	0.160	0.513
d 7	6.8	6.9	6.5	0.4	0.231	0.216
d 25	13.5	14.7	12.7	1.1	0.459	0.084
Average daily gain (g/d)						
Phase 1 (d 0 to 7)	1	21	−4	23	0.834	0.275
Phase 2 (d 7 to 25)	374	435	349	48	0.607	0.096
Overall (d 0 to 25)	270	320	250	39	0.617	0.095
Average daily feed intake (g/d)						
Phase 1 (d 0 to 7)	110	169	94	24	0.632	0.028
Phase 2 (d 7 to 25)	552	599	531	71	0.710	0.248
Overall (d 0 to 25)	422	466	390	55	0.469	0.121
Gain to feed ratio						
Phase 1 (d 0 to 7)	−0.17	−0.09	−0.30	0.31	0.753	0.702
Phase 2 (d 7 to 25)	0.63	0.74	0.67	0.04	0.543	0.112
Overall (d 0 to 25)	0.58	0.69	0.65	0.05	0.330	0.186

Each least squares mean represents 10 observations.

SEM, standard error of the mean.
